# New Genetic Determinants for qPCR Identification and the Enumeration of Selected Lactic Acid Bacteria in Raw-Milk Cheese

**DOI:** 10.3390/molecules29071533

**Published:** 2024-03-29

**Authors:** Milena Alicja Stachelska, Adam Ekielski, Piotr Karpiński, Tomasz Żelaziński, Bartosz Kruszewski

**Affiliations:** 1 Faculty of Computer Science and Technology, University of Lomza, Akademicka 14, 18-400 Lomza, Poland; pkarpinski@al.edu.pl; 2 Department of Production Engineering, Institute of Mechanical Engineering, Warsaw University of Life Sciences-SGGW, Nowoursynowska 164, 02-787 Warsaw, Poland; adam_ekielski@sggw.edu.pl (A.E.); tomasz_zelazinski@sggw.edu.pl (T.Ż.); 3 Department of Food Technology and Assessment, Institute of Food Sciences, Warsaw University of Life Sciences-SGGW, Nowoursynowska 159 C, 02-776 Warsaw, Poland

**Keywords:** cow’s milk cheese, qPCR, lactic acid bacteria, cheese ripening, genetic determinants, enumeration, molecular biology, protocol

## Abstract

Lactic acid bacteria (LAB) play an important role in the ripening of cheeses and contribute to the development of the desired profile of aroma and flavor compounds. Therefore, it is very important to monitor the dynamics of bacterial proliferation in order to obtain an accurate and reliable number of their cells at each stage of cheese ripening. This work aimed to identify and conduct a quantitative assessment of the selected species of autochthonous lactic acid bacteria from raw cow’s milk cheese by the development of primers and probe pairs based on the uniqueness of the genetic determinants with which the target microorganisms can be identified. For that purpose, we applied real-time quantitative PCR (qPCR) protocols to quantify *Lactobacillus delbrueckii* subsp. *bulgaricus*, *Streptococcus thermophilus*, and *Lactococcus lactis* subsp. *cremoris* cells in cheese directly after production and over three-month and six-month ripening periods. While *L. lactis* subsp. *cremoris* shows good acidification ability and the ability to produce antimicrobial compounds, *L. delbrueckii* subsp. *bulgaricus* has good proteolytic ability and produces exo-polysaccharides, and *S. thermophilus* takes part in the formation of the diacetyl flavor compound by metabolizing citrate to develop aroma, they all play an important role in the cheese ripening. The proposed qPCR protocols are very sensitive and reliable methods for a precise enumeration of *L. delbrueckii* subsp. *bulgaricus*, *S. thermophilus*, and *L. lactis* subsp. *cremoris* in cheese samples.

## 1. Introduction

Raw-milk cheese contains a microbiota that significantly contributes to the multiple varieties of the characteristics of ripened products through the variable dynamics of growth during processing. Different microorganisms, including bacteria, yeast, and mold, are simultaneously present in milk microbiota and many dairy products, especially those manufactured from raw milk. The problem lies in the fact that with such a wide variety of microorganisms present in cheese made from unpasteurized milk, there is a need to know which of these microorganisms make the greatest contribution to the quality of the finished product, which ones have the best growth rate, which of these microorganisms produce substances that inhibit the growth of potentially undesirable microorganisms from unpasteurized milk, and which of them are responsible for producing metabolites in the form of volatile compounds that give cheese the organoleptic characteristics highly desired by consumers. Lactic acid bacteria (LAB) constitute the indigenous and unique microflora of raw-milk cheeses and provide the organoleptic properties that distinguish such cheeses from their industrially mass-produced counterparts. This is the reason why it is worth studying the population growth dynamics of the selected lactic acid bacteria that ensure a high quality of ripened cheeses [1,2,3]. While *Lactococcus lactis* subsp. *cremoris* shows a good acidification ability and the ability to produce antimicrobial compounds, *Lactobacillus delbrueckii* subsp. *bulgaricus* has a good proteolytic ability and produces exo-polysaccharides, and *Streptococcus thermophilus* takes part in the formation of diacetyl flavor compounds by metabolizing citrate to develop aroma, they all play a huge role in cheese ripening [4]. Such characteristics of these microorganisms mean that it is reasonable to monitor the growth dynamics of the selected species of bacterial population during cheese ripening in order to understand the role of each microbial population [4,5].

Up to now, there are no precise methods to detect the selected species of LAB. The actual plate-counting methods give only a total amount of LAB, and they do not discern them by species. Plate-counting methods are methods that use microbiological media containing suitable nutrient compounds that enable the growth and multiplication of many different types of LAB. Thus, they do not provide a reliable qualitative and quantitative identification of specific LAB species. The plate-colony-counting methods have large operating errors, are usually time-consuming, and are characterized by low sensitivity [3,6]. In food matrices such as cheese, culture-independent methods have been rapidly recognized as a valuable alternative to culture-dependent methods [6,7,8,9,10]. Among culture-independent methods, quantitative real-time PCR (qPCR) represents a powerful tool for quantifying microbial populations. This molecular method allows simultaneous amplification and detection of specific DNA sequences. This method is recognized as rapid, specific, sensitive, and reliable. It enables the enumeration of microbial populations through the measurement of targeted gene copies. The method involves the extraction of nucleic acids and the amplification of the target region with specific primers/probe sets. The qPCR technology is based on the quantification of a target amplicon and one of the most precise variants when it is marked with a specific fluorescent probe [11,12,13,14]. 

In order to be able to know which strains are present in raw-milk cheese, we herein present a new method of quantification based on qPCR with primers/probe sets. The qPCR methods allow the specific quantification of different bacterial strains. 

The aim of this study was to identify and conduct a quantitative assessment of the selected species of autochthonous lactic acid bacteria from artisanal raw cow’s milk cheese. The novelty of this study relies on the development of primers/probe sets based on the uniqueness of the genetic determinants with which the target microorganisms can be identified. For that purpose, we applied qPCR assays for the quantification of *L. delbrueckii* subsp. *bulgaricus*, *S. thermophilus*, and *L. lactis* subsp. *cremoris*, which actively participate in milk fermentation and cheese ripening, to examine their growth and their ability to grow in cheese. The qPCR approach has not been previously used to study the proliferation dynamics of key lactic acid bacteria with extremely valuable properties that shape the appropriate aroma and flavor of ripened cheeses. As the number of publications on this issue is severely limited, the present study aims to fill this gap. 

## 2. Results

### 2.1. Assessing the Primers/Probe Sets Specificity

The specificity of the primers/probe sets was carried out with DNA hat was isolated from *L. delbrueckii* subsp. *bulgaricus*, *S. thermophilus*, and *L. lactis* subsp. *cremoris* strains and other bacteria to check if the newly designed primers/probe sets were specific only towards the examined lactic acid bacteria. The specificity of the primers and probes was tested with the other lactic acid bacteria as well as with less related species listed in Table 1. A more detailed informations can be found in Table A1. At first, the specificity of the primers/probe design was checked using the NCBI Primer-BLAST tool. Then, the specificity of the primers/probe sets was analyzed experimentally with gDNA that was isolated from different bacterial strains (Table 1). The sensitivity of the qPCR assays was conducted by preparing a serial tenfold dilution of the locus_tag=“LB080_06090”gene fragments of *L. delbrueckii* subsp. *bulgaricus*, the locus_tag “B1761_07395” gene fragments of *S. thermophilus*, and the locus_tag=“kw2_2318” gene fragments of *L. lactis* subsp. *cremoris,* which were treated as the positive controls. The dilutions were examined by qPCR, and the number of DNA copies contained in each dilution was used to calculate the limit of detection (LOD) and the linearity of the qPCR assay. The standard curve was created by plotting the Ct values of all dilutions as a function of the concentration of DNA copies and calculating the linear regression in R^2^. qPCR efficiency was assessed using standard curves with an equation PCR efficiency of (10^−1/slope^) − 1. *L. delbrueckii* subsp. *bulgaricus* genomes available in the GenBank database were searched for the dipeptidase production gene. All genomes were found to possess a single copy of this gene. *S. thermophilus* genomes available in the GenBank database were searched for the ATPase V production gene. All genomes were found to possess a single copy of this gene. *L. lactis* subsp. *cremoris* genomes available in the GenBank database were searched for the cation-transporting P-type ATPase gene. All genomes were also found to possess a single copy of this gene. The data obtained indicated that there was one copy of the genes in the genomes of the target microorganisms used for the quantitative detection.

*L. delbrueckii* subsp. *bulgaricus* DSM 20080, *S. thermophilus* APC 151, and *L. lactis* subsp. *cremoris* JM2 strains indicated average Cq (cycle threshold) values ± standard deviation amounting to 16.42 ± 0.09, 15.73 ± 0.09, and 15.77 ± 0.19, respectively (Table 1). The primer melting temperature (Tm) indicated values equal to 77.31 ± 0.09 °C for *L. delbrueckii* subsp. *bulgaricus*, 77.29 ± 0.14 °C for *S. thermophilus*, and 77.26 ± 0.11 °C for *L. lactis* subsp. *cremoris*, respectively (Table 2).

### 2.2. The qPCR Protocol Validation

**Figure 1:**
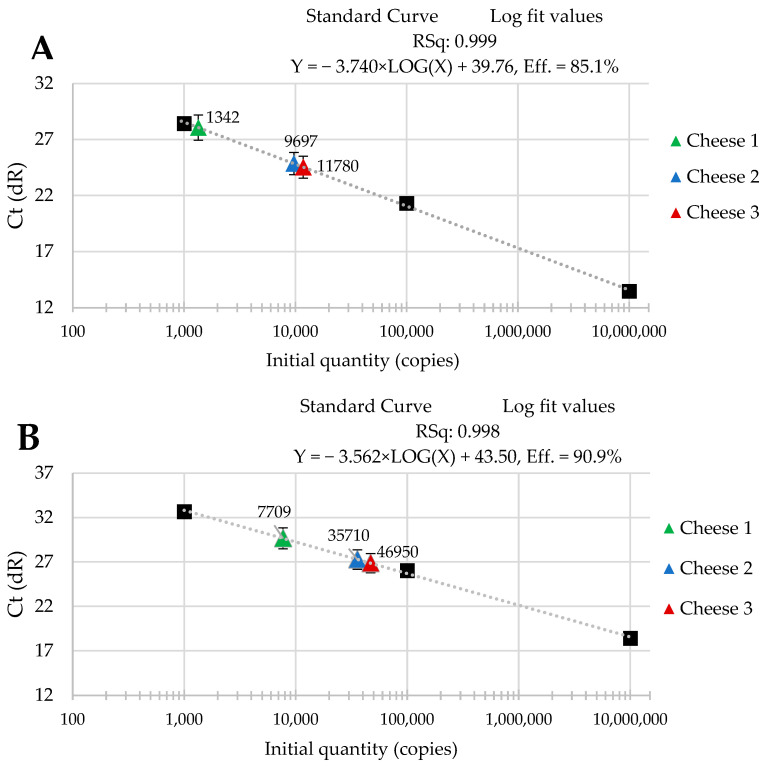

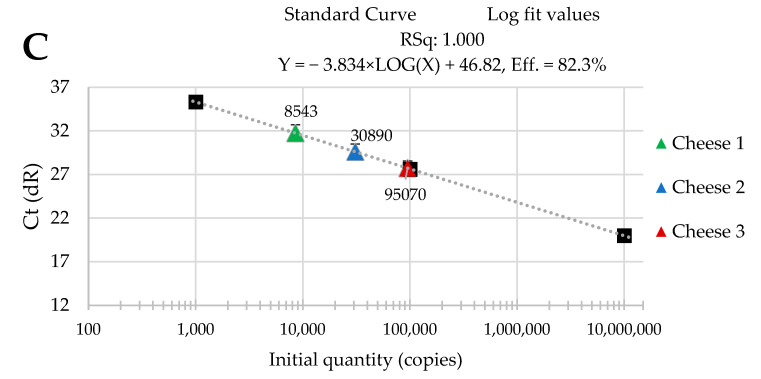
Performance of the qPCR protocol for quantitative detection of *L. delbrueckii* subsp. *Bulgaricus* in raw cow’s milk cheese (**A**) directly after production; (**B**) after 3-month ripening; (**C**) after 6-month ripening. A black square—content of gene copy present in 100-fold dilutions delineating the standard curve.

**Figure 2:**
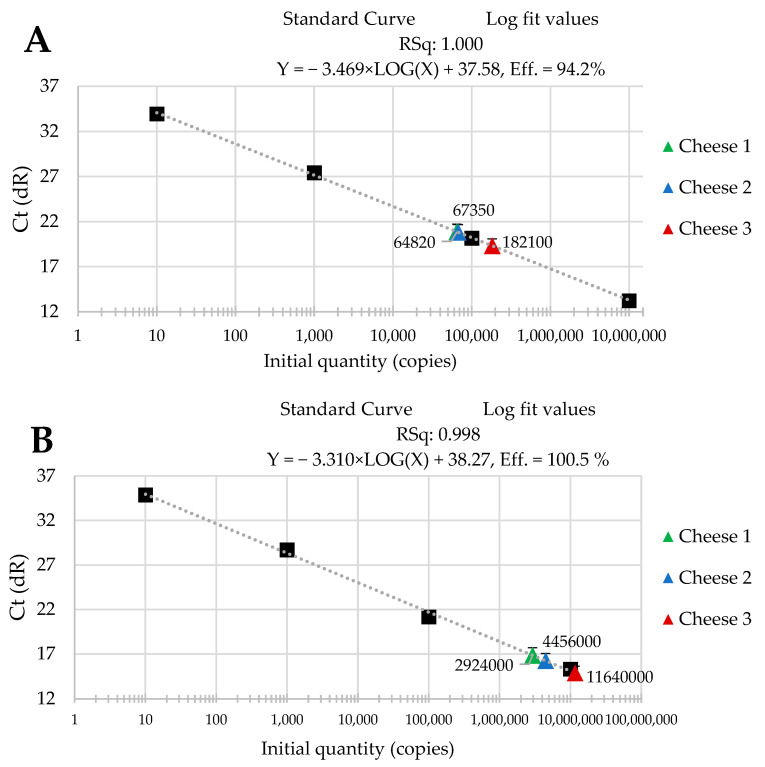

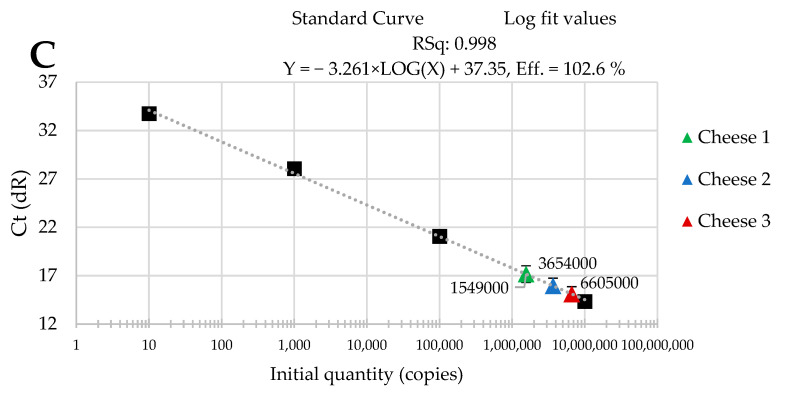
Performance of the qPCR protocol for quantitative detection of *S. thermophilus* in raw cow’s milk cheese (**A**) directly after production; (**B**) after 3-month ripening; (**C**) after 6-month ripening. A black square—content of gene copy present in 100-fold dilutions delineating the standard curve.

**Figure 3:**
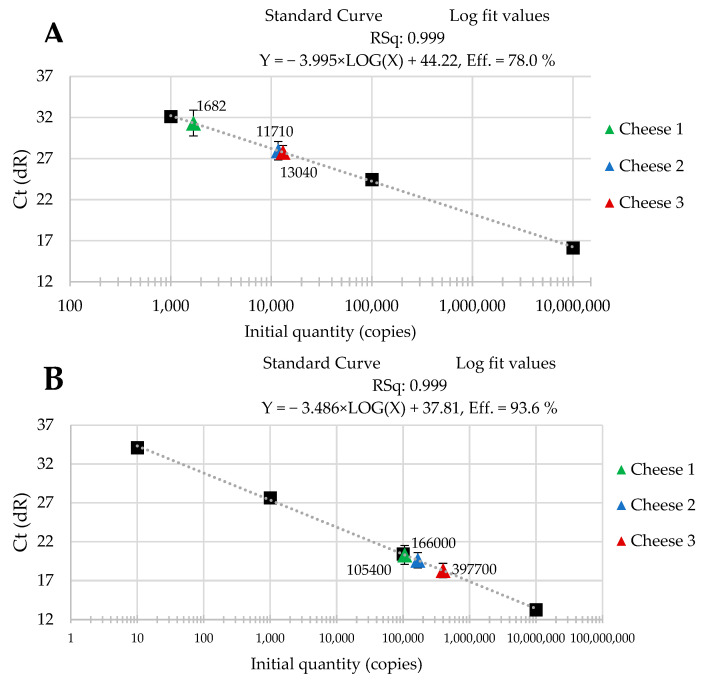

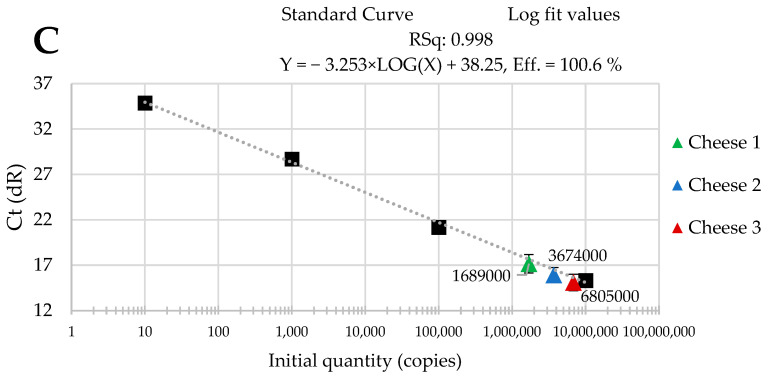
Performance of the qPCR protocol for quantitative detection of *L. lactis* subsp. *Cremoris* in raw cow’s milk cheese (**A**) directly after production; (**B**) after 3-month ripening; (**C**) after 6-month ripening. A black square—content of gene copy present in 100-fold dilutions delineating the standard curve.

The standard curves were received from tenfold serial dilutions of recombinant standard specific genes from *L. delbrueckii* subsp. *Bulgaricus* DSM 20080, *S. thermophilus* APC 151, and *L. lactis* subsp. *Cremoris* JM2, respectively. There was a good linear correlation between the Cq values and gene copy number/µL received for standard curves with R^2^ values that ranged from 0.998 to 1.000 for *L. delbrueckii* subsp. *Bulgaricus* (Figure 1A–C); from 0.998 to 1.000 for *S. thermophilus* (Figure 2A–C), and from 0.998 to 0.999 for *L. lactis* subsp. *Cremoris* (Figure 3A–C).

### 2.3. Enumeration of L. delbrueckii subsp. bulgaricus, S. thermophilus, and L. lactis subsp. cremoris in Cheese Samples by qPCR Method

Three batches of cheese produced from raw cow’s milk were examined to evaluate the usefulness of qPCR method for the enumeration of *L. delbrueckii* subsp. *bulgaricus*, *S. thermophilus*, and *L. lactis* subsp. *cremoris*. Cheese samples were analyzed immediately after cheese production and after three- and six-month ripening periods. Three qPCR protocols targeting specific genes were designed for the enumeration of *L. delbrueckii* subsp. *bulgaricus*, *S. thermophilus*, and *L. lactis* subsp. *cremoris* cells. New sets of primers/probe were developed, and their specificity was validated. The qPCR assays showed a high quantification capacity characterized by their linearity (R^2^ > 0.998) and PCR efficiencies were within the range 82.3–90.9% for *L. delbrueckii* subsp. *bulgaricus* (Figure 1A–C), 94.2–102.6% for *S. thermophilus* (Figure 2A–C), and 78.0–100.6% for *L. lactis* subsp. *cremoris* (Figure 3A–C). A properly designed assay, in the absence of interfering substances in the sample matrix, amplifies target DNA with at least 90% efficiency. In some reactions, the efficiency of the qPCR protocols was 85.1% (Figure 1A), 82.3 (Figure 1C), and 78% (Figure 3A). We isolated DNA from three different cheese batches. Cheese is a very difficult food matrix for DNA isolation due to its high fat content and high calcium ion content, which can inhibit the efficiency of the qPCR. This is probably the reason for the lower efficiency of qPCR in the three samples tested. In the remaining samples, qPCR efficiency was high and exceeded 90%.

**Figure 4:**
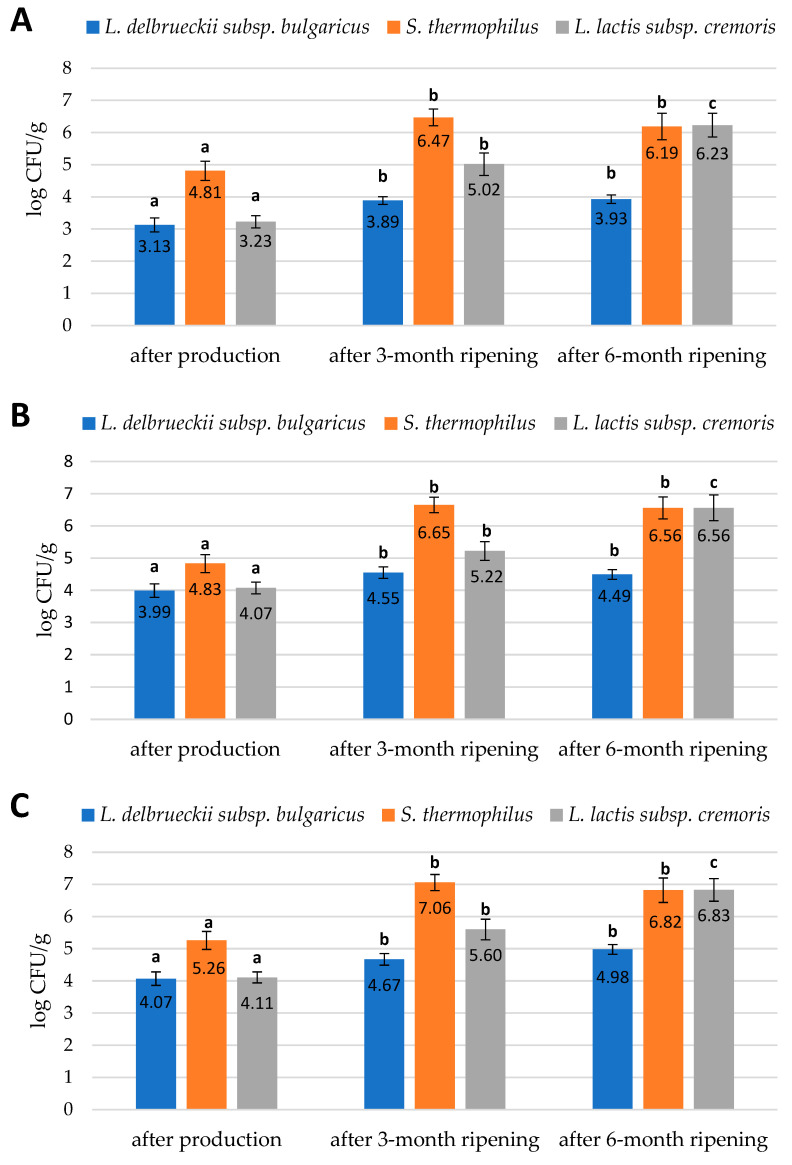
Numbers of *L. delbrueckii* subsp. *bulgaricus*, *S. thermophilus*, and *L. lactis* subsp. *cremoris* cells in (**A**) the first batch of cheese; (**B**) the second batch of cheese; (**C**) the third batch of cheese; expressed in log CFU/g obtained by qPCR method. The bars of the same color marked with different letters are significantly different (*p* < 0.05).

The data that we obtained during our study are presented in Figure 1, Figure 2 and Figure 3. They reveal the performance of the qPCR protocol for quantitative detection of the target microorganisms. The numbers of the colony-forming units in 1 g of cheese are expressed as a number of copies of the particular gene characteristic for a given target microorganism. The data presented in Figure 1, Figure 2 and Figure 3 show how the numbers of the three target microorganisms, namely *L. delbrueckii* subsp. *bulgaricus*, *S. thermophilus*, and *L. lactis* subsp. *cremoris*, changed during ripening in three different batches of cheese starting from production and after three- and six-months periods of ripening. The data are presented as the number of copies of a given gene specific only to a given species per gram of cheese. In order to check how many copies of a specific gene were present in the genome of the target microorganisms, we used the NCBI Blast Nucleotide database. All the genomes of the target microorganisms were found to possess a single copy of a specific gene. Thus, the data obtained proved that there was one copy of the genes in the genomes of the target microorganisms used for the quantitative detection, which means that the number of copies of a particular gene is actually the number of colony-forming units of a particular target species present in 1 g of cheese, which resulted from searching genomes using the NCBI Blast Nucleotide database. In order to make the results shown in Figure 1, Figure 2 and Figure 3 easier to read, we present them graphically in Figure 4A–C, where we show the dynamics of proliferation of the target microorganisms in the cheeses from the time the cheeses were produced and after three- and six-months periods of ripening. We converted CFU/g to log CFU/g for better clarity.

Three batches of cheese made from unpasteurized cow’s milk were tested in three repetitions. The research was conducted using the qPCR method to quantify a number of selected lactic acid bacteria present in three batches of cheese immediately after production and after 3 and after 6 months of ripening. The three batches of cheese were characterized by slightly differentiated numbers of the target species. The numbers of target bacteria belonging to the species *L. delbrueckii* subsp. *bulgaricus*, *S. thermophilus*, and *L. lactis* subsp. *cremoris* found in the cheeses at different ripening stages are shown in Figure 4A–C. The error bars in Figure 4A–C are a graphical representation of the variability in the data, and they are used in graphs to indicate an error or uncertainty in the reported measurement. They give a general idea of how accurate a measurement is or, conversely, how far from the reported value the true value can be.

The study showed that immediately after the production of the cheese batches, bacteria belonging to the *L. delbrueckii* subsp. *bulgaricus* species were present in the lowest numbers in relation to *S. thermophilus* and *L. lactis* subsp. *cremoris* bacteria. The first batch of cheese tested immediately after production had the lowest number of *L. delbrueckii* subsp. *bulgaricus* and *L. lactis* subsp. *cremoris* bacteria compared to the second and third batches. In contrast, the population size of *S. thermophilus* was the highest in the third batch of cheese after production. Nevertheless, these differences were not significant. Milk for the production of these three batches of cheese came from three different farms in the Podlasie region; hence, there were some differences in the population size of the microorganisms studied. After three months of ripening, the number of *S. thermophilus* and *L. lactis* subsp. *cremoris* bacteria in all three cheese batches tested increased significantly. A less significant increase was observed for the *L. delbrueckii* subsp. *bulgaricus* population. After six months of ripening, there was a significant increase in the population of *L. lactis* subsp. *cremoris* and a nonsignificant increase in the population of *L. delbrueckii* subsp. *bulgaricus*, while the population of *S. thermophilus* was slightly reduced (Figure 4A–C).

## 3. Discussion

Our aim was to evaluate the number of the selected species of LAB in the ripened cheese by applying the qPCR method because the existing plate-colony-counting methods have some problems, as they do not provide an accurate assessment of the number of a particular type of LAB, and they are time-consuming [10]. Thus, we focused our efforts on the search and assessment of new genetic determinants for target LAB screening. We applied the qPCR method, providing the rapid qualitative and quantitative detection of three very important LAB that have a significant influence on the cheese-ripening process.

It is important to know what types of bacteria are present in cheeses made from unpasteurized milk and what role they have. Cheeses made from unpasteurized milk can potentially contain undesirable microorganisms [15]. Cheeses, due to their high nutrient content, high water content, use of low-temperature heat treatment, and lack of preservative additives, may be a good environment for the growth of various microorganisms, including potentially dangerous pathogens that may pose a health risk to consumers. It should be emphasized that lactic acid bacteria constitute the indigenous microflora of cheeses made from unpasteurized milk [16,17,18,19,20,21]. Lactic acid bacteria have very good acidifying and flavor-forming properties. Furthermore, some LAB strains have been recognized as potentially probiotic microorganisms. Some LAB counteract the proliferation of pathogenic bacteria by producing lactic acid and producing antimicrobial peptides such as bacteriocins [22,23]. Given that lactic acid bacteria are capable of producing aromatic and unique metabolic compounds, cheeses made with them are particularly preferred by consumers due to the presence of a large amount of volatile compounds responsible for the palatability of the final product. There is also a tendency to isolate specific indigenous strains of LAB from cheeses made from unpasteurized milk for the production of starters used for the manufacture of cheeses made from pasteurized milk in dairy plants [24,25,26,27,28].

Lactic acid bacteria that have very good acidification and aroma-forming properties and inhibit the growth of undesirable microflora in raw-milk cheeses include *L. delbrueckii* subsp. *bulgaricus*, *S. thermophilus*, and *L. lactis* subsp. *cremoris.* They are Gram-positive lactic acid bacteria and play a significant role in dairy fermentation. Coppola et al. [15] investigated the numbers of *S. thermophilus* in cheese during ripening. The number of live cells decreased during ripening. Blaiotta et al. [16] examined lactobacilli (*L. helveticus* and *L. delbrueckii* ssp. and *lactis*) that were present in industrial starter cultures for processing hard cheese (e.g., Emmental, Comté, Italian Grana, and Argentinean hard cheeses). Coppola et al. [17] examined a number of *L. delbrueckii* ssp. *bulgaricus* in Pasta filata cheeses (mozzarella) and Italian hard cheeses (Canestrato Pugliese and Parmigiano Reggiano) [16,18]. Chamba [19] found that the number of lactobacilli decreased during ripening of Emmental cheese. The decrease was dependent on the sensitivity of lactobacilli to salt, on the water activity, and on the autolysis power of the strains. 

Some lactobacilli were also investigated as bacteria present in the dairy products produced from raw cow’s milk [16,20]. They were found to exist initially in small numbers (10^2^ to 10^3^/g directly after pressing in cheddar cheese); then, they increased to significantly high numbers in cheeses that need long ripening times (10^7^ to 10^8^ bacteria/g after 3-month ripening in cheddar cheese) [21]. Coppola et al. [17] examined the microflora of raw milk, natural whey starter, and cheese during the first months of ripening of Parmigiano Reggiano. It was found that thermophilic lactobacilli rapidly decreased in number during one month of ripening. On the other hand, mesophilic facultatively heterofermentative lactobacilli progressively increased in number during five months of ripening [22,23,24,25].

Previous studies have shown that the use of different strains of lactic acid bacteria resulted in differences in the quantity and quality of flavor and aroma compounds, which is of particular importance in the production of traditional products [26]. The quality and repeatability of cheeses and the process of their production is guaranteed in the industry by the use of starter cultures; however, consumers prefer products produced in a traditional way, e.g., by natural methods of milk acidification, which ensure that better sensory characteristics are obtained as a result [27]. The cheese owes its unique and specific characteristics to the type of raw material used in its production, the microbiological quality of raw material, the degree of acidification of milk, the production technology, and the ripening conditions [28]. Raw-milk cheeses are considered to be rich in taste and are part of the cultural heritage of many countries. Different microorganisms, including bacteria, yeast, and mold, are simultaneously present in milk microbiota and many dairy products, especially those manufactured from raw milk [25]. This microbiota greatly contributes to the broad diversity of the characteristics of similar ripened products through variable dynamics during processing. These dynamics are largely influenced by interactions between microorganisms, e.g., microbial co-operation and antagonism, and have a marked impact on the survival, growth, and activity of the different microbial populations during the cheese production process [26]. It is therefore of primary importance to reliably quantify physiologically active populations, in terms of dynamic changes, in order to understand the role of each microbial population. LABs are the most commonly used bacteria in the production of fermented products. They not only have the ability to produce lactic acid but also produce specific compounds responsible for shaping the flavor, aroma, and texture characteristics of cheese. They carry out proteolysis and lipolysis by metabolizing proteins and fats [28]. The LABs undergo the autolysis and release intracellular enzymes involved in the transformation of various compounds during the ripening of cheese. LABs also produce antimicrobial compounds such as organic acids, diacetyl, acetoin, hydrogen peroxide, and bacteriocin. Bacteriocins are peptides with antimicrobial properties [29]. The positive effect of the LABs on the quality of cheese is due to the synthesis of compounds that give it a specific, identifying aroma. The most important volatile compounds formed by lactic fermentation bacteria are diacetyl, acetaldehyde, acetic acid, and ethanol. Other compounds such as fatty acids, alcohols, acetone, and esters are produced in much smaller quantities. The presence of such compounds in cheese improves safety, controls the fermentation process, accelerates ripening, and extends the shelf life of the product [30,31]. Thus, monitoring the number of selected lactic acid bacterial species during cheese ripening makes it possible to obtain a final product with excellent flavor and aroma characteristics as well as a product of high microbiological quality [20,31]. The results of our study indicated that the lowest numbers of the three different target microorganism cells of *L. delbrueckii* subsp. *bulgaricus*, *S. thermophilus*, and *L. lactis* subsp. *cremoris* were found in three batches of cheese immediately after production. Bacteria belonging to the *S. thermophilus* species were most abundant in all three batches of cheese immediately after production. Their number increased significantly after three months of ripening and remained at a comparable level for a further three months of ripening, showing a slightly decreasing trend. The number of bacteria of the *L. lactis* subsp. *cremoris* species remained at a similar level to that of the *L. delbrueckii* subsp. *bulgaricus* species immediately after production. The number of bacteria belonging to the *L. lactis* subsp. *cremoris* species increased moderately after three months of ripening and still increased after six months of ripening, becoming equal to the population of *S. thermophilus* after six months of ripening. Our results showed the lowest log increase in the *L. delbrueckii* subsp. *bulgaricus* bacterial population after six months of ripening. The results show a moderate logarithmic increase in the *L. lactis* subsp. *cremoris* bacterial population after three months of ripening in the three cheese batches tested and a dynamic logarithmic increase in the population of these bacteria after six months of ripening in relation to the initial amount immediately after production. The bacterial population belonging to the *S. thermophilus* species had the highest growth rate in the first three months of ripening (Figure 4A–C). Our study showed no significant differences in the evaluation results of the three cheese batches. The similar trends in the proliferation rate of target microorganisms in each of the three cheese batches tested probably result from the fact that milk for the production of these cheeses came from three neighboring farms with the same breed of cows and using a very similar feeding program for their cows.

The efficiency of our qPCR assays is not always the same. We are aware that the most common reasons for lower efficiencies are bad primers–probe design and non-optimal reaction conditions. Secondary structures like dimers and hairpins or inappropriate melting temperatures (Tm) might affect primer–template annealing, which results in poor amplification. In order to prevent such a situation, we used the Beacon Designer software (http://www.premierbiosoft.com/qpcr/) to analyze the secondary structure of the designed probe and primers. We checked the accuracy of the designed probe in terms of melting temperature, percentage of cytosine and guanine, and formation of cross dimers, self-dimers, and hairpins. We checked the specificity of the probe and primers in the NCBI BLAST database. We obtained satisfactory results. We also analyzed the purity of the isolated DNA samples with spectrophotometric measurements prior to qPCR. Purity was measured as the ratio of absorbance values at 260 and 280 nm, which correspond to the ratio of nucleic acids to other molecules. Despite the fact that the purity score did not fall below 1.8 for DNA, we obtained too low a qPCR efficiency in some reactions. The material from which we isolated DNA consisted of three different batches of cheese. Cheese is a difficult material for DNA isolation due to its high fat content and high calcium ion content, which can inhibit the efficiency of the PCR reaction. Thus, the higher the fat content of the examined material, the more difficult the DNA extraction procedure. The composition of dairy products greatly influences the efficiency of DNA extraction. The likely reason for obtaining too low a qPCR efficiency in some reactions was the necessity to isolate DNA from a very difficult material such as cheese, with its high fat content.

## 4. Materials and Methods

### 4.1. Cheese Production

For experimental purposes, three batches of artisanal cheese were made based on the traditional method using unpasteurized cow’s milk of very high microbiological quality. Milk used to make the cheese came from the selected organic farms of Podlasie region in Poland. Full-fat, unpasteurized cow’s milk with a fat content of 4.8% was heated to a temperature of 32 °C, and then, salt and rennet were added, causing the milk to coagulate within thirty minutes. The resulting curd was cut into cubes of about 5 cm thick and reheated to 42 °C. The cheese mass thus formed was placed in colanders to drain off the whey and carry out the acidification process of the cheese mass at 22 °C for 48 h. After 2 days of maturing, we obtained fresh cheese, after which we removed it from the colanders, rubbed the mass with salt, and placed the shaped cheeses on shelves in a maturing chamber at 12 °C. After maturing for 10 days, the cheese turned yellow in color and became slightly firmer on the surface and considerably drier. Its flavor was distinctive, with a slightly nutty note. The ripened form of the artisanal cheese was obtained after 6 months of ripening. We studied the multiplication rate of the selected three strains of lactic acid bacteria immediately after production as well as after 3 and 6 months of ripening in a ripening chamber at a temperature of 12 °C and air humidity of 80–85%.

### 4.2. DNA Extraction from the Cheese Samples

The study material was microbial DNA from cheese made from unpasteurized milk, which was isolated immediately after production and after three and six months of ripening. DNA was isolated from cheese by using Syngen Food DNA Mini Kit (Syngen Biotech, Wrocław, Poland). A 200 mg sample of cheese was weighed out. The AXIS ATA220 electronic laboratory balance with the precision of 0.001 g (Superwagi, Warsaw, Poland) with an internal calibration was used for precise cheese weighing. Cheese in an amount of 200 mg of the homogenized sample was placed in 2 mL tube; then, 1 mL of buffer DLF (2% cetyl trimethylammonium bromide (CTAB) (*w*/*v*), 1% polyvinylpyrrolidone-40 (PVP40) (*w*/*v*), 2% β-mercaptoethanol (*v*/*v*)) was added. The tube was closed and mixed by vortexing. Next, 30 µL of proteinase K (Bioron GmbH, Römerberg, Germany) was added; the tube was closed and mixed by vortexing and then incubated at 60 °C for 30 min. Then, it was centrifuged, and 700 µL of the supernatant was transferred to a new 2 mL tube. In some food samples, the three phases can be formed, which is due to the presence of various proteins contained in the cheese matrix, including casein and whey proteins. Then, 500 µL of chloroform was added, and the tube was closed and vortexed. Next, 350 µL of the upper phase was transferred to a new 2 mL tube, and then, 350 µL of buffer DWF (3 M guanidine chloride, 70% isopropyl alcohol (*v*/*v*)) was added. The column DF was placed in a 2 mL tube. All the material was transferred into the column DF. The tube was then centrifuged. The supernatant was discarded, and the column was transferred back to the tube. Next, 700 μL of buffer DPF (70% ethanol (*v*/*v*)) was added to the column. The column was centrifuged. The supernatant was discarded, and the column was transferred back to the tube. The column was centrifuged and transferred to a new 1.5 mL tube. Then, 100–200 μL of pre-warmed DE elution buffer (0.1 M Tris-ethylenediaminetetraacetic acid (EDTA), pH 7.5) was added at the center of the membrane and incubated at room temperature for 1 min. DE elution buffer helps protect resuspended DNA against degradation caused by pH changes that occur during freeze–thaw cycles. The lid was closed, and the tube was centrifuged for 1 min at maximum speed (18,000× *g*). The variation in DNA extraction efficiency was checked by the measurement of concentrations of extracted DNA. DNA concentrations were assessed by measuring absorbance using the spectrophotometer (Evolution 220, Thermo Fisher Scientific, Waltham, MA, USA), and the purity was evaluated based on its absorbance at 260–280 nm. An A_260_/A_280_ ratio of 1.8–2.0 characterizes high DNA quality. The extracted DNA had an A_260_/A_280_ ratio amounting to 1.8–2.0, which means it had high quality and purity, and the high efficiency of the DNA extraction procedure from the produced cheese was confirmed.

### 4.3. Designing Probes and Primers for Real-Time PCR Assays

GSMer software https://github.com/qichao1984/GSMer (accessed on 15 November 2023) was used to identify genome-specific markers (GSMs) from currently sequenced microbial genomes using a k-mer-based approach. A link to a file with the characteristic nucleotide sequences of the target microorganisms was downloaded from this program http://ieg.ou.edu/GSMer/allgsm_species.zip (accessed on 15 November 2023) and then opened with the tool http://glogg.bonnefon.org/download.html (accessed on 15 November 2023). The Excel file (http://ieg.ou.edu/GSMer/strain.xlsx (accessed on 15 November 2023)) was used to find the target species with their assigned signatures. The sequence file was then used to find the characteristic nucleotide sequences of the target microorganisms’ genes using their assigned signatures. NCBI Blast Nucleotide software (https://blast.ncbi.nlm.nih.gov/Blast.cgi (accessed on 15 November 2023)) was then used to find the sequence region of the characteristic gene, and then, primers and a probe were designed based on this region. Nucleotide sequences of the primers/probes sets used for the detection of *L. delbrueckii* subsp. *bulgaricus*, *S. thermophilus*, and *L. lactis* subsp. *cremoris* are shown in Table 2. The nucleotide primers/probe sets’ sequences for each lactic acid bacterial species are also provided in Table A1. The sequence of the genes were provided by GenBank (www.ncbi.nlm.nih.gov/Genbank/ accessed on 15 November 2023; Accession Numbers: CP019120.1, CP019935.1, and CP015900.1). The nucleotide sequences of the locus_tag=“LB080_06090” gene (localized from the nucleotide position 1,188,025 to 1,189,443 in the complete genome) that encodes the dipeptidase production unique to *L. delbrueckii* subsp. *bulgaricus* were compared with those of closely related strains. The nucleotide sequences of the locus _tag “B1761_07395” gene (localized from the nucleotide position 1,349,595 to 1,350,980 in the complete genome) that encodes the ATPase V production unique to *Streptococcus thermophilus* were compared with those of closely related strains. The nucleotide sequences of the locus_tag=“kw2_2318” gene (localized from the nucleotide position 2,391,233 to 2,393,926 in the complete genome) that encodes the “cation-transporting P-type ATPase” production unique to *Lactococcus lactis* subsp. *cremoris* were compared with those of closely related strains. These are the genes from which the amplicons were obtained. 

The primers/probe sets were designed using Beacon Designer Software v8.0 (Premier Biosoft International, San Francisco, CA, USA). The sets were validated using NCBI BLAST (Basic Local Alignment Search Tool: www.ncbi.nlm.nih.gov/blast/ (accessed on 15 November 2023)). Thereby, the 60 bp region from the nucleotide position 1,188,781 to 1,188,841 within the locus_tag=“LB080_06090” gene was found to be a potential target site because it was identical in all examined *L. delbrueckii* subsp. *bulgaricus* strains but indicated variability in other LAB species. 

The 60 bp region from the nucleotide position 1,349,701 to 1,349,761 within the locus _tag “B1761_07395” gene was found to be a potential target site because it was identical in all examined *S. thermophilus* strains but indicated variability in other LAB species.

The 60 bp region from the nucleotide position 2,393,641 to 2,393,701 within the locus _tag “LLJM2_2339” gene was found to be a potential target site because it was identical in all examined *L. lactis* subsp. *cremoris* strains but indicated variability in other LAB species. The TagMan probes had a FAM reporter dye at its 5′ end and an MGB-NFQ quencher dye at its 3′ end. The Beacon Designer v. 8 software was used to check the characterization of the probe designs in terms of Tm, GC content, and the free enthalpy of the decay reaction of the most stable II-row structure (deltaG).

The oligonucleotides were synthesized and bought from Eurofins Genomics (Germany). The fluorescence was detected using an optical detection system incorporated in the thermocycler of Stratagene Mx3005P (Real-Time PCR Detection System, Agilent Technologies, Santa Clara, CA, USA).

### 4.4. Bacterial Strains and Culture Conditions

The primer specificity test was carried out with the bacterial strains presented in Table 1. The concentration of bacterial cell culture was measured by spectrophotometer (Evolution 220, Thermoscientific, Waltham, MA, USA). *L. delbrueckii* subsp. *bulgaricus* was propagated in 10 mL of MRS broth at 42 °C, and *S. thermophilus* and *L. lactis* subsp. *cremoris* were propagated in 10 mL of M17 broth at 42 °C. Cells coming from a 2 mL late exponential growth-phase culture (A_650 nm_ = 0.7–0.8) were collected by centrifugation at 3000× *g* for 5 min at 4–6 °C and stored at −20 °C until DNA extraction. Then, 1 mL of enrichment culture was pipetted into a 2 mL microcentrifuge screw-cap tube and was centrifuged at 13,000× *g* for 5 min. Then, the supernatant was discarded using a pipette. Care was taken to not disrupt the pellet. Then, 200 µL of Fast Lysis Buffer (Syngen Biotech, Frankfurt, Germany) was added to the bacterial pellet, the tube was tightly capped, and the pellet was resuspended by vigorous vortexing. Then, the microcentrifuge tube was placed into a thermal shaker (800× *g*) set to 100 °C. The sample was heated for 10 min. The sample was removed and cooled to room temperature (15–25 °C) for 2 min. The tube was centrifuged at 13,000× *g* for 5 min. After centrifugation, the supernatant was carefully transferred to a new tube, and 2 µL of this supernatant was used as the template.

### 4.5. Preparation of Standard Curves on the Basis of Solutions of Gene Fragments

The sequences of the genes were provided by GenBank (www.ncbi.nlm.nih.gov/Genbank/ (accessed on 15 November 2023); Accession Numbers: CP019120.1, CP019935.1, and CP004884.1, respectively). They were used to prepare serial dilutions of gene copies coming from *L. delbrueckii* subsp. *bulgaricus*, *S. thermophilus*, and *L. lactis* subsp. *cremoris*, respectively. A total of 1.27 × 10^12^ DNA copies were dissolved in 1270 µL of DE buffer (Syngen Biotech, Wrocław, Poland), achieving a concentration of 1 × 10^9^ DNA copies/µL of eluate. The locus_tag=“LB080_06090”gene fragments of *L. delbrueckii* subsp. *bulgaricus*, the locus _tag “B1761_07395” gene fragments of *S. thermophilus*, and the locus_tag=“kw2_2318” gene fragments of *L. lactis* subsp. *cremoris* were cloned into plasmids, synthesized, and purchased from Eurofins Genomics (Ebersberg, Germany). They were delivered in the lyophilized form. Standard curves were prepared with serial dilutions of the sequence of the genes of the selected lactic acid bacteria. They were dissolved in 1270 µL of DE buffer (Syngen Biotech, Wrocław, Poland), achieving a concentration of 1 × 10^9^ DNA copies/µL of eluate. This concentration was used for the preparation of standards for standard curve. The dilutions were prepared to achieve 10^1^ DNA copies/µL of eluate in the highest dilution. A tenfold dilution series of the PCR fragment solution for each bacterial species, covering 7 logs ranging from 10^1^ to 10^7^ DNA copies per reaction, was used to estimate the sensitivity of the method. 

### 4.6. The Real-Time PCR Conditions

The volume of reaction mixture amounted to 20 μL. Real-time PCR analysis was carried out using Stratagene Mx3005P thermocycler (Agilent Technologies, Santa Clara, CA, USA). The PCR reaction mixture consisted of 5 μL (10 ng/μL) of DNA template; 4 μL of Quantum Probe Mix (Syngen Biotech, Cambridge, UK); 0.8 μL (0.4 μM) of primers F and R, respectively; 0.5 μL (0.25 μM) of hydrolysis probe; and 8.9 μL of PCR water. A non-template control (NTC) contained 5 µL of water instead of DNA and was included in each run. The real-time PCR cycling parameters were as follows: 1 cycle of amplification (95 °C for 5 min) and 35 cycles of amplification (94 °C for 30 s, 60 °C for 30 s, and 72 °C for 90 s). The fluorescence of the reporter dye (FAM) was measured during the amplification at 510 nm. The real-time PCR reaction and amplification step were performed using the DNA amplification curves being the subject of analysis. The calculation of the threshold cycle (C_T_) value was performed with the use of the Stratagene Mx3005P software version 2.1 (Agilent Technologies, Santa Clara, CA, USA). The C_T_ value was described as the real-time PCR cycle, at which the generated fluorescence increased exponentially and exceeded its background level. The standard curves were created by plotting the threshold cycle (C_T_) values as a function of the concentration of recombinant “LB080_06090”standard gene copies of *L. delbrueckii* subsp. *bulgaricus* per µL, the locus _tag “B1761_07395” standard gene copies of *S. thermophilus* per µL, and the locus_tag=“kw2_2318” standard gene copies of *L. lactis* subsp. *cremoris* per µL. All standard and sample reactions were run in triplicate. The qPCR inhibition was checked using two basic approaches. The first method analyzed the quantification cycle (Cq) deviation of a spiked internal positive control. The second method considered variations in reaction efficiency based on the slopes of individual amplification plots. By combining those methods, we did not observe increased Cq values together with reduced amplification efficiencies.

### 4.7. Statistical Analysis

All measurements were performed in triplicate. Statistical analyses such as one-way ANOVA with Tukey’s test, calibration curves, and coefficients of determination were performed using Sigma plot v. 11 software (Systat Software, San Jose, CA, USA).

## 5. Conclusions

The proposed qPCR protocols are highly effective methods for accurate enumeration of the key lactic acid bacteria involved in cheese ripening. The cheeses examined in this study were made from unpasteurized milk, the microflora composition of which is highly variable compared to cheeses made from pasteurized milk with the addition of starter cultures, with an always-repeatable composition. However, cheeses made from unpasteurized milk have much richer taste and aroma qualities and are therefore highly desired by consumers. Our results show that certain lactic acid bacteria increase their numbers during different cheese ripening stages, while others decrease their numbers. Compared with the existing methods, the method developed in this study allows for a quantitative assessment of the selected species of autochthonous lactic acid bacteria from raw cow’s milk cheese to be conducted by the development of primers/probe sets based on the uniqueness of the genetic determinants with which the target microorganisms can be identified. We developed new genetic determinants for target LAB screening. Our newly developed detection systems were found to be very sensitive and reliable for a precise enumeration of *L. delbrueckii* subsp. *bulgaricus*, *S. thermophilus*, and *L. lactis* subsp. *cremoris* in the cheese samples.

## Figures and Tables

**Table 1:** The Cq values achieved from qPCR protocols using DNA isolated from *L. delbrueckii* subsp. *bulgaricus*, *S. thermophilus*, and *L. lactis* subsp. *cremoris* (constituting the positive controls) and the closely related bacterial species (constituting negative controls).

Name of the Tested Bacteria ^1^	*Lactobacillus* *delbrueckii* subsp. *bulgaricus* Primer–Probe Pairs	*Streptococcus* *thermophilus* Primer–Probe Pairs	*Lactococcus lactis* subsp. *cremoris* Primer–Probe Pairs	Mean Cq Value in Positive Control
*Lactobacillus delbrueckii* subsp. *bulgaricus* DSM 20080	Positive control	Negative control	Negative control	16.42 ± 0.09
*Lactobacillus delbrueckii* subsp. *bulgaricus* ATCC 11842	Positive control	Negative control	Negative control	16.70 ± 0.13
*Lactobacillus delbrueckii* subsp. *bulgaricus* ATCC BAA-365	Positive control	Negative control	Negative control	16.51 ± 0.09
*Lactobacillus delbrueckii* subsp. *bulgaricus* L99	Positive control	Negative control	Negative control	15.56 ± 0.08
*Lactobacillus delbrueckii* subsp. *bulgaricus* LBA-40	Positive control	Negative control	Negative control	15.76 ± 0.11
*Lactococcus lactis* subsp. *cremoris* JM2	Negative control	Negative control	Positive control	15.77 ± 0.19
*Lactococcus lactis* subsp. *cremoris* A76	Negative control	Negative control	Positive control	16.35 ± 0.15
*Lactococcus lactis* subsp. *cremoris* MG1363	Negative control	Negative control	Positive control	15.83 ± 0.26
*Lactococcus lactis* subsp. *cremoris* NZ9000	Negative control	Negative control	Positive control	15.86 ± 0.29
*Streptococcus thermophilus* APC 151	Negative control	Positive control	Negative control	15.73 ± 0.09
*Streptococcus thermophilus* ATCC 19258	Negative control	Positive control	Negative control	15.37 ± 0.11
*Streptococcus thermophilus* CS18	Negative control	Positive control	Negative control	16.35 ± 0.09
*Streptococcus thermophilus* APC 151	Negative control	Positive control	Negative control	16.39 ± 0.12
*Streptococcus thermophilus* CS5	Negative control	Positive control	Negative control	15.78 ± 0.05
*Streptococcus thermophilus* ST3	Negative control	Positive control	Negative control	15.97 ± 0.13
*Lactococcus lactis* subsp. *lactis* 14B4	Negative control	Negative control	Negative control	nd ^2^
*Lactococcus lactis* subsp. *lactis* F44	Negative control	Negative control	Negative control	nd
*Lactococcus lactis* subsp. *lactis* UL8	Negative control	Negative control	Negative control	nd
*Lactobacillus plantarum* HFC8	Negative control	Negative control	Negative control	nd
*Lactobacillus plantarum* 022AE	Negative control	Negative control	Negative control	nd
*Lactobacillus plantarum* W2	Negative control	Negative control	Negative control	nd
*Lactobacillus rhamnosus* ATCC 11443	Negative control	Negative control	Negative control	nd
*Lactobacillus rhamnosus* LR5	Negative control	Negative control	Negative control	nd
*Lactobacillus rhamnosus* LMG 23550	Negative control	Negative control	Negative control	nd
*Escherichia coli* NCCP_14540	Negative control	Negative control	Negative control	nd
*Escherichia coli* 2014_3057	Negative control	Negative control	Negative control	nd
*Escherichia coli* EPSSH018	Negative control	Negative control	Negative control	nd
*Escherichia coli* STEC411	Negative control	Negative control	Negative control	nd
*Pseudomonas fluorescens* SBW25	Negative control	Negative control	Negative control	nd
*Pseudomonas fluorescens* L321	Negative control	Negative control	Negative control	nd
*Pseudomonas fluorescens* NZ007	Negative control	Negative control	Negative control	nd

^1^ 10 ng of DNA; ^2^ nd, not detected with the Ct value > 40.

**Table 2:** Nucleotide sequences of the primers/probe sets used for the detection of *L. delbrueckii* subsp. *bulgaricus*, *S. thermophilus*, and *L. lactis* subsp. *cremoris*.

Bacteria/Target Gene	Tm Value (°C)	qPCR Efficiency	Primers/ Probe	Primers/Probe Sets’ Sequences (5′ → 3′)	Accession Number Provided by GenBank
*Lactobacillus delbrueckii* subsp. *bulgaricus*/ locus _tag “LB080_06090” gene	77.23	82.3–90.9%	Forward primer	GCTTGTTTTTGGCAACAA	CP019120.1
	77.41		Reverse primer	AGTTGACTTTACTAGTGA	
	77.29		Probe	GACTTCATGTGGAGTGCAGGGATCCAGGAC	
*Streptococcus thermophilus*/ locus _tag “B1761_07395” gene	77.14	94.2–102.6%	Forward primer	TAACAAGACGTCCCATGGT	CP019935.1
	77.33		Reverse primer	GTGTGTCAGCTATTGCTAC	
	77.41		Probe	GTGTCTATGGATCTGACTTCTAGTCTTAAC	
*Lactococcus lactis* subsp. *cremoris*/locus _tag “LLJM2_2339” gene	77.15	78.0–100.6%	Forward primer	TTGCTTCTACGATTAAT	CP004884.1
	77.26		Reverse primer	TAGTTGTAATTCCTCTTG	
	77.38		Probe	GAAGAGAGATTCATTACTTCTAGCGTTATAG	

## Data Availability

Data are contained within the article.

## Appendix A

**Table A1:** The Tm values and primers/probe sets’ sequences used for qPCR analysis results presented in Table 1.

Bacteria Sample ^1^	Tm Value (°C)	Primers/ Probe	Primers/Probe Sets’ Sequences (5′→ 3′)	Accession Number Provided by GenBank
*Lactobacillus delbrueckii* subsp. *bulgaricus* DSM 20080	77.23	Forward primer	GCTTGTTTTTGGCAACAA	CP019120.1
	77.41	Reverse primer	AGTTGACTTTACTAGTGA	
	77.29	Probe	GACTTCATGTGGAGTGCAGGGATCCAGGAC	
*Lactobacillus delbrueckii* subsp. *bulgaricus* ATCC 11842	77.33	Forward primer	GCTTGTTTTTGGCAACAA	CR954253.1
	77.81	Reverse primer	AGTTGACTTTACTAGTGA	
	77.51	Probe	GACTTCATGTGGAGTGCAGGGATCCAGGAC	
*Lactobacillus delbrueckii* subsp. *bulgaricus* ATCC BAA-365	77.51	Forward primer	GCTTGTTTTTGGCAACAA	CP000412.1
	77.38	Reverse primer	AGTTGACTTTACTAGTGA	
	77.62	Probe	GACTTCATGTGGAGTGCAGGGATCCAGGAC	
*Lactobacillus delbrueckii* subsp. *bulgaricus* L99	78.21	Forward primer	GCTTGTTTTTGGCAACAA	CP017235.1
	78.32	Reverse primer	AGTTGACTTTACTAGTGA	
	78.29	Probe	GACTTCATGTGGAGTGCAGGGATCCAGGAC	
*Lactobacillus delbrueckii* subsp. *bulgaricus* LBA-40	78.12	Forward primer	GCTTGTTTTTGGCAACAA	CP102529.1
	78.24	Reverse primer	AGTTGACTTTACTAGTGA	
	78.18	Probe	GACTTCATGTGGAGTGCAGGGATCCAGGAC	
*Lactococcus lactis* subsp. *cremoris* KW2	77.15	Forward primer	TTGCTTCTACGATTAAT	CP004884.1
	77.26	Reverse primer	TAGTTGTAATTCCTCTTG	
	77.38	Probe	GAAGAGAGATTCATTACTTCTAGCGTTATAG	
*Lactococcus lactis* subsp. *cremoris* A76	77.12	Forward primer	TTGCTTCTACGATTAAT	CP003132.1
	77.29	Reverse primer	TAGTTGTAATTCCTCTTG	
	77.18	Probe	GAAGAGAGATTCATTACTTCTAGCGTTATAG	
*Lactococcus lactis* subsp. *cremoris* MG1363	78.49	Forward primer	TTGCTTCTACGATTAAT	AM406671.1
	77.81	Reverse primer	TAGTTGTAATTCCTCTTG	
	78.12	Probe	GAAGAGAGATTCATTACTTCTAGCGTTATAG	
*Lactococcus lactis* subsp. *cremoris* NZ9000	77.39	Forward primer	TTGCTTCTACGATTAAT	CP002094.1
	78.61	Reverse primer	TAGTTGTAATTCCTCTTG	
	77.32	Probe	GAAGAGAGATTCATTACTTCTAGCGTTATAG	
*Streptococcus thermophilus* APC 151	78.31	Forward primer	TAACAAGACGTCCCATGGT	CP019935.1
	78.51	Reverse primer	GTGTGTCAGCTATTGCTAC	
	78.42	Probe	GTGTCTATGGATCTGACTTCTAGTCTTAAC	
*Streptococcus thermophilus* ATCC 19258	77.62	Forward primer	TAACAAGACGTCCCATGGT	CP038020.1
	78.48	Reverse primer	GTGTGTCAGCTATTGCTAC	
	77.47	Probe	GTGTCTATGGATCTGACTTCTAGTCTTAAC	
*Streptococcus thermophilus* CS18	77.51	Forward primer	TAACAAGACGTCCCATGGT	CP030928.1
	77.42	Reverse primer	GTGTGTCAGCTATTGCTAC	
	77.27	Probe	GTGTCTATGGATCTGACTTCTAGTCTTAAC	
*Streptococcus thermophilus* APC 151	77.14	Forward primer	TAACAAGACGTCCCATGGT	CP019935.1
	77.33	Reverse primer	GTGTGTCAGCTATTGCTAC	
	77.41	Probe	GTGTCTATGGATCTGACTTCTAGTCTTAAC	
*Streptococcus thermophilus* CS5	78.23	Forward primer	TAACAAGACGTCCCATGGT	CP028896.1
	78.43	Reverse primer	GTGTGTCAGCTATTGCTAC	
	78.48	Probe	GTGTCTATGGATCTGACTTCTAGTCTTAAC	
*Streptococcus thermophilus* ST3	78.35	Forward primer	TAACAAGACGTCCCATGGT	CP017064.1
	78.28	Reverse primer	GTGTGTCAGCTATTGCTAC	
	78.38	Probe	GTGTCTATGGATCTGACTTCTAGTCTTAAC	
*Lactococcus lactis subsp. lactis* 14B4	nd ^2^	Forward primer	TAAAGGAACCGAAGACG	CP028160.1
	nd	Reverse primer	GATGATGATTCTTGTGGA	
	nd	Probe	TAATTACTGACGGTTCTATCGTTCACGTTT	
*Lactococcus lactis subsp. lactis* F44	nd	Forward primer	TAAAGGAACCGAAGACG	CP024954.1
	nd	Reverse primer	GATGATGATTCTTGTGGA	
	nd	Probe	TAATTACTGACGGTTCTATCGTTCACGTTT	
*Lactococcus lactis subsp. lactis* UL8	nd	Forward primer	TAAAGGAACCGAAGACG	CP015908.1
	nd	Reverse primer	GATGATGATTCTTGTGGA	
	nd	Probe	TAATTACTGACGGTTCTATCGTTCACGTTT	
*Lactobacillus plantarum* HFC8	nd	Forward primer	GTTGCGGACTTTCAAGTGT	CP012650.1
	nd	Reverse primer	TCCGTGACCAGATAACCA	
	nd	Probe	CGGGTCAGCGCTCTGACTAGTTGGTTGACG	
*Lactobacillus plantarum* 022AE	nd	Forward primer	GTTGCGGACTTTCAAGTGT	CP031127.2
	nd	Reverse primer	TCCGTGACCAGATAACCA	
	nd	Probe	CGGGTCAGCGCTCTGACTAGTTGGTTGACG	
*Lactobacillus plantarum* W2	nd	Forward primer	GTTGCGGACTTTCAAGTGT	CP091094.1
	nd	Reverse primer	TCCGTGACCAGATAACCA	
	nd	Probe	CGGGTCAGCGCTCTGACTAGTTGGTTGACG	
*Lactobacillus rhamnosus* ATCC 11443	nd	Forward primer	TTGCTGGATTCGCCTACTC	CP022109.1
	nd	Reverse primer	AGTACCGTCTGAGGTGT	
	nd	Probe	ATTGAACCCAAGTAAGGGTTGTAGATTTGA	
*Lactobacillus rhamnosus* LR5	nd	Forward primer	TTGCTGGATTCGCCTACTC	CP017063.1
	nd	Reverse primer	AGTACCGTCTGAGGTGT	
	nd	Probe	ATTGAACCCAAGTAAGGGTTGTAGATTTGA	
*Lactobacillus rhamnosus* LMG 23550	nd	Forward primer	TTGCTGGATTCGCCTACTC	CP136119.1
	nd	Reverse primer	AGTACCGTCTGAGGTGT	
	nd	Probe	ATTGAACCCAAGTAAGGGTTGTAGATTTGA	
*Escherichia coli* NCCP_14540	nd	Forward primer	AGCTCTGAATTAAGGGCC	CP042982.1
	nd	Reverse primer	ACTGGCGCTGACAATTGT	
	nd	Probe	GCCGCGTCCGCATCTCCATTACCGTCTTAT	
*Escherichia coli* 2014_3057	nd	Forward primer	AGCTCTGAATTAAGGGCC	CP027387.1
	nd	Reverse primer	ACTGGCGCTGACAATTGT	
	nd	Probe	GCCGCGTCCGCATCTCCATTACCGTCTTAT	
*Escherichia coli* EPSSH018	nd	Forward primer	AGCTCTGAATTAAGGGCC	CP125889.1
	nd	Reverse primer	ACTGGCGCTGACAATTGT	
	nd	Probe	GCCGCGTCCGCATCTCCATTACCGTCTTAT	
*Escherichia coli* STEC411	nd	Forward primer	AGCTCTGAATTAAGGGCC	CP061243.1
	nd	Reverse primer	ACTGGCGCTGACAATTGT	
	nd	Probe	GCCGCGTCCGCATCTCCATTACCGTCTTAT	
*Pseudomonas fluorescens* SBW25	79.45	Forward primer	CTCTCCGTACGTCACTGTA	OV986001.1
	79.23	Reverse primer	AGCGAGGTTCGAAGAAAA	
	78.67	Probe	TCTCCCGAGAGGTACACAATCTGATCGCGGA	
*Pseudomonas fluorescens* L321	78.37	Forward primer	CTCTCCGTACGTCACTGTA	CP015637.1
	79.38	Reverse primer	AGCGAGGTTCGAAGAAAA	
	78.62	Probe	TCTCCCGAGAGGTACACAATCTGATCGCGGA	
*Pseudomonas fluorescens* NZ007	nd	Forward primer	CTCTCCGTACGTCACTGTA	OV986001.1
	nd	Reverse primer	AGCGAGGTTCGAAGAAAA	
	nd	Probe	TCTCCCGAGAGGTACACAATCTGATCGCGGA	

^1^ 10 ng of DNA; ^2^ nd, not detected with the Cq value > 40.
